# Effect of tropical forest disturbance on the competitive interactions within a diverse ant community

**DOI:** 10.1038/s41598-018-23272-y

**Published:** 2018-03-23

**Authors:** Ross E. J. Gray, Robert M. Ewers, Michael J. W. Boyle, Arthur Y. C. Chung, Richard J. Gill

**Affiliations:** 1 Department of Life Sciences, Imperial College London, Silwood Park Campus, Buckhurst Road, Ascot, SL5 7PY UK; 2 Forest Research Centre, Forestry Department, P.O. Box 1407, 90715 Sandakan, Sabah Malaysia

## Abstract

Understanding how anthropogenic disturbance influences patterns of community composition and the reinforcing interactive processes that structure communities is important to mitigate threats to biodiversity. Competition is considered a primary reinforcing process, yet little is known concerning disturbance effects on competitive interaction networks. We examined how differences in ant community composition between undisturbed and disturbed Bornean rainforest, is potentially reflected by changes in competitive interactions over a food resource. Comparing 10 primary forest sites to 10 in selectively-logged forest, we found higher genus richness and diversity in the primary forest, with 18.5% and 13.0% of genera endemic to primary and logged respectively. From 180 hours of filming bait cards, we assessed ant-ant interactions, finding that despite considered aggression over food sources, the majority of ant interactions were neutral. Proportion of competitive interactions at bait cards did not differ between forest type, however, the rate and per capita number of competitive interactions was significantly lower in logged forest. Furthermore, the majority of genera showed large changes in aggression-score with often inverse relationships to their occupancy rank. This provides evidence of a shuffled competitive network, and these unexpected changes in aggressive relationships could be considered a type of competitive network re-wiring after disturbance.

## Introduction

Species assemblage has been realised as a major determinant of ecosystem processes and function^1,2^, therefore, it is important to understand how the leading threats to biodiversity, such as anthropogenic disturbance, can alter community composition^3–5^. Human-modified landscapes have increasingly become a part of many ecosystems^6^ and mounting evidence supports that disturbance alters the interactive processes within communities and subsequent ecological function^4,7,8^. Understanding the effects of disturbance requires deep knowledge of how community composition changes^9,10^, and to link end-point patterns of change with the reinforcing interactive processes that structure communities^11,12^.

Competition between taxonomic and/or functional groups is considered an important interactive process determining community composition and structure^13,14^, and it is reasonable to presume that habitat disturbance will alter these interactions^15^. Indeed, studies on plant communities have shown that persistent anthropogenic disturbance can cause seemingly irreversible shifts in grassland competitive communities^16,17^, and the defoliation of dominant plant species can decrease interspecific competitive interaction intensity and importance^18^. Furthermore, community interaction network studies have reported a re-wiring of interactions following disturbance in soil microbes^19,20^, and following the removal of plants or pollinators in mutualistic plant-pollinator community networks^21–23^. However, experiments attempting to elucidate how the dynamics of competitive interactions across multi-node networks change after disturbance are limited^12,24^. Here, we aim to contribute to this paucity of data by examining how potential changes in the community composition of ants, found between undisturbed and disturbed sites of a lowland dipterocarp rainforest in Borneo, is reflected in a change to the observed competitive interactions over a food resource between the nodes of the ant community network.

*Ant community in a disturbed forest of Borneo*: The Indo-Malayan region of the tropics is a global hotspot of biodiversity^25^, but worryingly is experiencing some of the highest rates of forest loss compared with other tropical realms^26–28^. With nearly 80% of the land surface on Borneo having been affected by logging and oil palm conversion^29,30^, it is important we understand how communities are responding to such change^8^. Ants constitute an estimated 25% of animal biomass in tropical forests^31^, and play a number of critical functional roles in ecosystem processes, often acting as ecosystem engineers^32,33^. The ubiquity, functionality and competitive structuring in ant communities may make ants some of the most cost-effective indicators of tropical forest disturbance^34–36^.

Competition among ants has been recognized as a key process in the structuring of their communities^24,37–42^, with interactions between certain taxonomic groups, when competing over a common resource, often showing consistent uni-directional aggressive behaviour(s) from one group to the other^43,44^. It has been proposed that such patterns in behaviour can dictate ant spatial distributions (occupancy)^40,45,46^, abundances^47^ and potentially even species richness^48,49^. Importantly, the removal of competitive groups in a community may create competitive release, changing the abundance or distribution of other groups by allowing them to newly access and exploit previously denied resources^50–52^. The abundance and/or occupancy of a taxonomic/functional group in the community is thus likely to be related to components of their competitive ability over other constituent groups. In other words, a change in frequency of one competitive node is likely to have a cascading or ripple effect across the competitive network, rather than just a ‘one-step effect’ on only the directly linked nodes.

*Testing the influence of disturbance on ant community composition and competition*: The effect of anthropogenic forest disturbance on animal communities, such as ants, has been of interest to ecologists^38,53–57^, but despite their critical functional roles and apparent importance of competition for structuring their communities, studies specifically examining the effect of disturbance on competitive interactions within communities are relatively lacking. Here, we conducted a study that first investigated the difference in ant community composition between primary and selectively-logged sites in an extensive Bornean forest. Primary forest acted as the control because it had not undergone anthropogenic forest disturbance in the form of logging or clearing for agriculture^58^. Selectively-logged forest acted as the disturbed forest comparison, as the removal of specific trees from a forest area results in canopy gaps, destroyed undergrowth, and the presence of extensive road networks^59^. In identifying both the similarities and differences in community composition between the sites, we then set out to observe inter-genus interactions at bait cards, allowing us to explore how ant abundance and occupancy relates to variation in competitive interactions for the shared ant genera between the forest types. As a proxy for competitive ability we observed each genus-genus interaction on a bait card and scored whether it was an aggressive, neutral or submissive response for each individual. In the absence of prior experiments of this nature, we tested a null hypothesis in which aggression-scores among genera observed in primary forest would be the same in logged forest, regardless of any change in relative abundances or occupancy between forest types. Alternatively, changes in abundance or occupancy could positively correlate with aggression-score (intuitive hypothesis) causing a shuffling of dominance in the competitive network, as variations to the presence of numerically dominant ants has been shown to increase interspecific aggression in a previous study^60^.

## Methods

### Study Area

Study sites were situated in the Malaysian state of Sabah, on the island of Borneo, at the site of the Stability of Altered Forest Ecosystems project^58^. We established 10 control (undisturbed) sites in primary lowland dipterocarp rainforest within the Maliau Basin Conservation Area (4°44″N, 116°58″E), and another 10 disturbed sites located in selectively-logged lowland dipterocarp rainforest within the Ulu Segama Forest Reserve (4°43″N, 117°35″E). The primary forest had never been logged and has an average aboveground tree biomass of 350 t.ha^−1^, in contrast to the selectively-logged forest which was logged in the 1970’s and again in the 1990’s, removing an estimated 228 t.ha^−1^ of tree biomass^61^. The logged forest was characterized by a more open canopy with lower leaf area index and smaller trees^61^. The choice to use the Stability of Altered Forest Ecosystems project as a research location was important, as the design and location of study sites is considered to minimise the confounding factors that affect land-use change, such as latitude, slope and elevation^58^.

### Sampling Design

**Figure 1:**
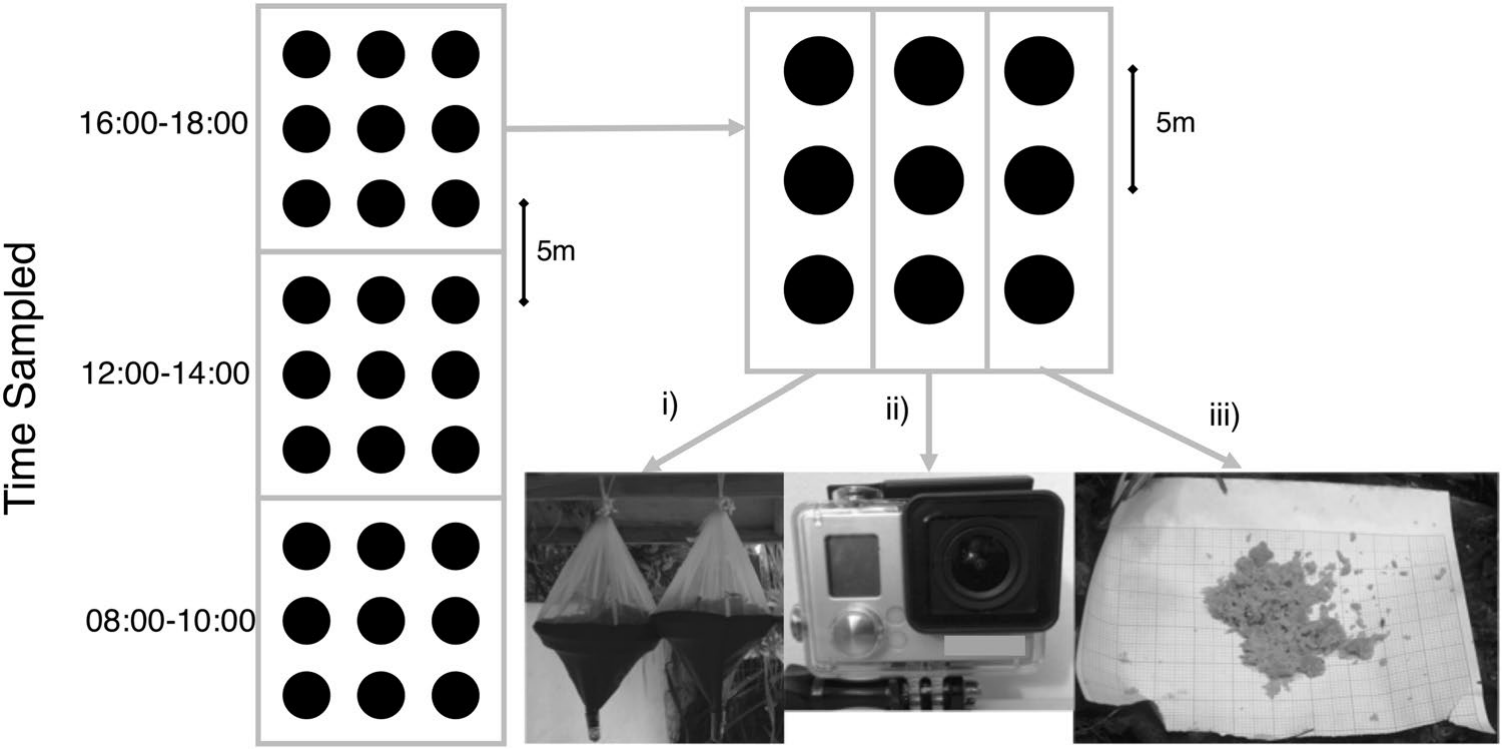
Sampling design used at each site in primary and selectively-logged forest. Black circles represent sampling points. (**i**) Winkler bag extraction method in which all leaf litter across all three sampling points in the column was pooled. (**ii**) Camera set-up above a bait card to record 40 mins of ant interactions at each point in the column. (**iii**) Human observation for 40 mins of a bait card placed at each sampling point in the column, where all observed ants were attempted to be collected using an aspirator. Photo credits: (**i**) Kirsty Yule; (**ii** & **iii**) Ross Gray.

Field data focused on sampling ground dwelling ants collected between February-June 2016. An initial sampling site in each forest type was chosen at random, and the remaining nine sites were spaced sequentially along a transect at intervals of 60–100 m to ensure independence. At each sampling site, 27 sampling points were established using a 3 × 9 grid pattern design (composed of three sets of 3 × 3 connected grids) with each sampling point spaced 5 m from its nearest neighbour (Fig. 1). Due to previously established differences in ant activity at different times of day^62,63^, ants were sampled for 2 hours from the first of the grids at 0800 h, the second grid at 1200 h and last grid at 1600 h.

#### Assessing community composition between forest types

Each of the three grids at a sample site was split into three columns, each with a different sampling method (Fig. 1). We used both active and passive traps in combination, allowing us to collect both fast and slow-moving genera^63–65^.

- For column 1, ant individuals were collected, and genus identified from leaf litter samples. We scraped up and collected all leaf litter within a 1 × 1 m quadrat at each sampling point^66^. Leaf litter collected from the three sampling points within each grid was pooled and placed into a single Winkler bag^67^ (an extraction method that separates live specimens from dead vegetation; Fig. 1(i)). Each bag was left to hang for 3 days, a period shown to collect up to 90% of the ant species present in the sample^68^, with ants collected in a vial containing 70% ethanol to be later identified (total = 30 bags per forest type).
- For column 2, a bait card was placed at each sampling point, with each card consisting of a sheet of graph paper (210 × 148.5 mm) placed flush with the ground and baited with two heaped teaspoons of a tuna and cat-food mix (mixed at a 15:1 weight ratio) (Fig. 1(ii)). Ant genera were identified visiting a bait card by non-invasively re-watching recordings from a GoPro™ video camera (Model: HERO3 White Edition, specifications in Supplementary Information) that was placed above the card for 40 mins per bait card (justification provided in Supplementary Information). Bait cards have been widely used to study food exploitation by ants^69–71^. To attract a range of ant genera we used tuna as a protein bait and cat food provides a carbohydrate nutrition base both of which have been highly effective in attracting ants in previous studies^70,72–74^. Data from only the video recordings were used to investigate ant competitive interactions.
- For column 3, we again used a bait card, but this was monitored for 40 mins by a human observer and any ants that crawled on to the card were invasively hand collected, placed in 70% ethanol and later identified (Fig. 1(iii)). Collection was used to build a reference collection and to provide a search image to help identify ant genera from the column 2 video footage. This data was not used for the community composition analysis or competitive interactions because pilot study data indicated observer presence could disrupt ant presence and interactions.

Ants collected from columns 1 and 3 were identified to genus level under a stereo microscope with the help of a taxonomic identification key^75^ and photographs from online databases^76,77^. We used genus level identification because this is an efficient method of taxonomic sorting that ant community composition studies have used previously^78–80^. Moreover, ants can be separated into functional groups at the genus level^79–82^, and importantly these functional groups have been shown to display differing levels of dominance/aggression^79,81,83^. Given this, we felt that genus-level identification was sufficient to begin to unpick the competitive mechanisms within such a highly diverse ant community.

#### Assessing competitive interactions among genera

From the column 2 video recordings, we noted the time and order of arrival for each genus, and any direct interactions with individuals belonging to other ant genera; processes which have been shown to affect competitive ability in previous studies^60,70,73^. Interactions were categorized as neutral or competitive: *neutral* interactions were when the individuals of one genus did not change the behaviour of individuals in the other genus; *competitive* interactions were when individuals of one genus showed aggression toward individuals of another genus^69,70^. For all competitive interactions, genera were further classified as either *aggressive*, where individuals of that genus showed aggression and/or forced individuals of the other to retreat from the bait; or *submissive*, where an individual fled from the bait when confronted by another from a different genus.

Rate of competitive interactions were defined as the total number of competitive interactions over the 40 min observation, and per capita number of competitive interactions was calculated by dividing the total number of interactions by the total abundance of ants on a bait card over the 40 min observation period. We assigned an aggression-score to each genus involved in a pairwise genus-genus interaction (0 = Submissive; 1 = Neutral; 2 = Aggressive), and then calculated a mean score for each pairwise genus-genus combination. Consequently, we had multiple aggression-scores for each genus – one for each of the other genera that the target genus interacted with. Only genera that had interactions with >3 other genera were analysed. Scores were produced separately for interactions observed in the primary and logged forest, and the difference between forest-specific scores were calculated to represent an aggression-score change. Considering unequal representation of the genera in samples because of colony and sampling method variations, we weighted the aggression scores based on their sample size to encompass the variability. The mean of the differences in a genus’ aggression-score between forest types were also calculated to represent a change in an aspect of their competitive status between primary and logged forest. The mean of this competitive change was then compared to changes in occupancy to investigate whether variations in competitive interactions are associated with variations in community composition.

### Data analysis

All statistical analyses were carried out in R (R Development Core Team^84^).

#### Community Composition

A Generalised Linear Model (GLM) with quasipoisson error distribution was used to examine the effect of forest type on genus richness. Mean Shannon diversity (*H’*) was also calculated and a linear regression used to compare ant genera community evenness between forest types^85^. Occupancy refers to the number of sampling points in which the focal genus was present at out of all sampling points (120 per site). We used occupancy rather than abundance to reduce any sampling bias that could be introduced if sampling was near or far away from a colony (nest) entrance^86^. Further Mann-Whitney U tests were used to compare the occupancy of genera in each sampling method. We used a Spearman’s rank correlation to compare changes to the rank occupancy of genera between forest types.

#### Competitive Interactions

We used Mann-Whitney U tests to examine the ratio of neutral to competitive interactions in primary and logged forests, and we used a GLM with binomial error distribution to test the effect forest type had on this ratio. To assess whether ant interactions changed between forest type we employed a mixed-effects model (GLMER) using the R package lme4^87^. To compare the level of competitive interactions between forest types, the interactions were categorised into single binary response variables: 1 = Competitive (where between two individuals one is dominant, and one is submissive), 0 = Neutral (where between two individuals both are neutral). GLMERs were also used to examine the effect of forest type on the proportion, the rate and the per capita number of competitive interactions at each bait card. The random effect of *site*, containing 10 levels per forest type, was included in the models to account for the nested structure of our sampling design. Following this, we used a Spearman’s rank correlation to compare changes in aggression-score with changes in occupancy rank between forest types.

## Results

### Variation in community composition between forest types

**Figure 2:**
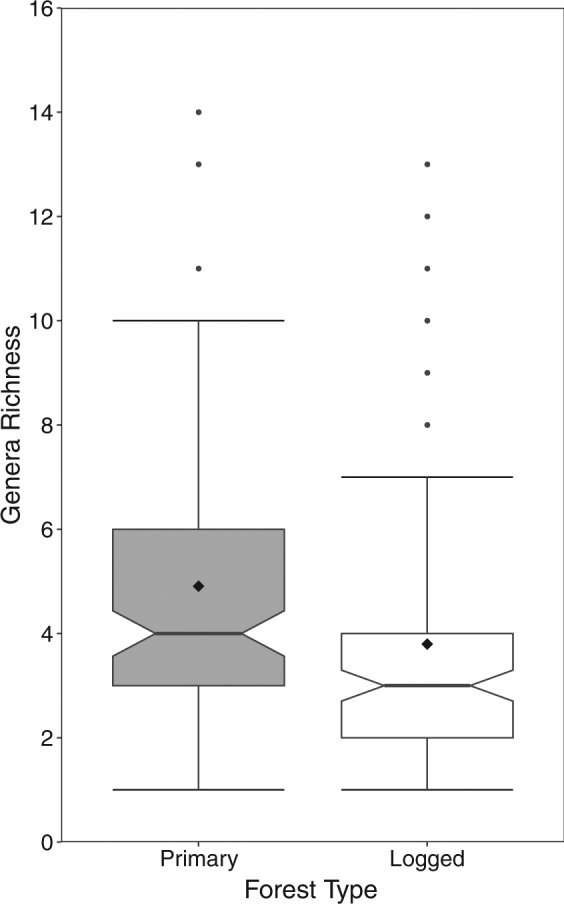
Comparison of mean genera richness between primary and logged forest (*P* = 0.001). Dissecting line and black diamond represents the median and mean, respectively, boxes represent the inter-quartile ranges, whiskers show 95% CI and dots show outliers.

The combined leaf litter and bait card data identified 54 genera, with 32 identified exclusively from the leaf litter, three exclusively observed visiting the bait traps, and 19 identified using both collection methods. The two forest types had 68.5% (*N* = 37) of genera in common, with 18.5% (*N* = 10) being exclusive to primary forest and the remaining 13.0% (*N* = 7) exclusive to logged forest (Fig. S1b). Taking the mean genus richness across 120 sampling points per forest type (n = 30 leaf litter + 90 camera observations), we found primary forest had a significantly higher richness than logged forest (4.91 vs. 3.80; GLM: Z_229_ = 3.258, *P* = 0.001; Fig. 2; Table S1). Furthermore, primary forest displayed a significantly higher community evenness in comparison to logged forest (Mean *H’*: Primary = 2.03, Logged = 1.84; t_118_ = 2.759, *P* = 0.007; Table S1).

**Figure 3:**
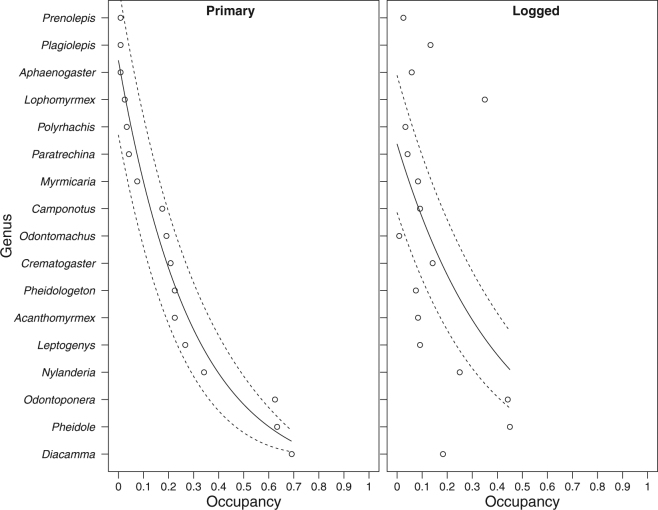
Rank occupancy plot for the 17 competitive genera found in both forest types, with primary forest compared with the same rank, plotted against logged forest. Genera are ordered by decreasing rank occupancy in primary forest up the y axis. The solid line represents a generalised linear regression (GLM) prediction using quasipoisson error distribution. Dashed lines represent the 95% confidence intervals for the GLM line.

Of the 22 genera that were observed to interact, 17 had interactions with >3 other genera and so these were focused on for the analysis. A plot of the rank occupancy of these 17 genera showed a change in community composition between forest types, due primarily to an increase in occupancy of *Lophomyrmex* and a decrease in occupancy of *Diacamma, Pheidole* and *Odontoponera* (*S* = 368, Rho = 0.55, *P* = 0.022; Fig. 3). Communities in both habitat types were dominated by *Odontoponera* and *Pheidole*, despite both genera showing small reductions in their occupancy.

### Difference in competitive interactions among genera between forest types

We observed 615 interactions among 22 of the 54 genera identified. When standardising the amount of time observed in each forest type, we saw three times more interactions in primary than in logged forest (489 and 146 respectively), despite very little difference in total ant abundance in the two forest types (Primary: 1659 vs. Logged: 1622). One hundred and sixty-three interactions were competitive and 452 neutral. Interestingly, whilst a significantly higher proportion of neutral interactions were found in primary forest (Competitive: 120, Neutral: 369; W = 1, *P* = 0.012) this was not seen to the same degree in logged (Competitive: 43, Neutral: 83; W = 18.5, *P* = 0.061), with the difference between forest types close to being significantly different (GLM: Z_17_ = −1.89, *P* = 0.059; Table S1).

**Figure 4:**
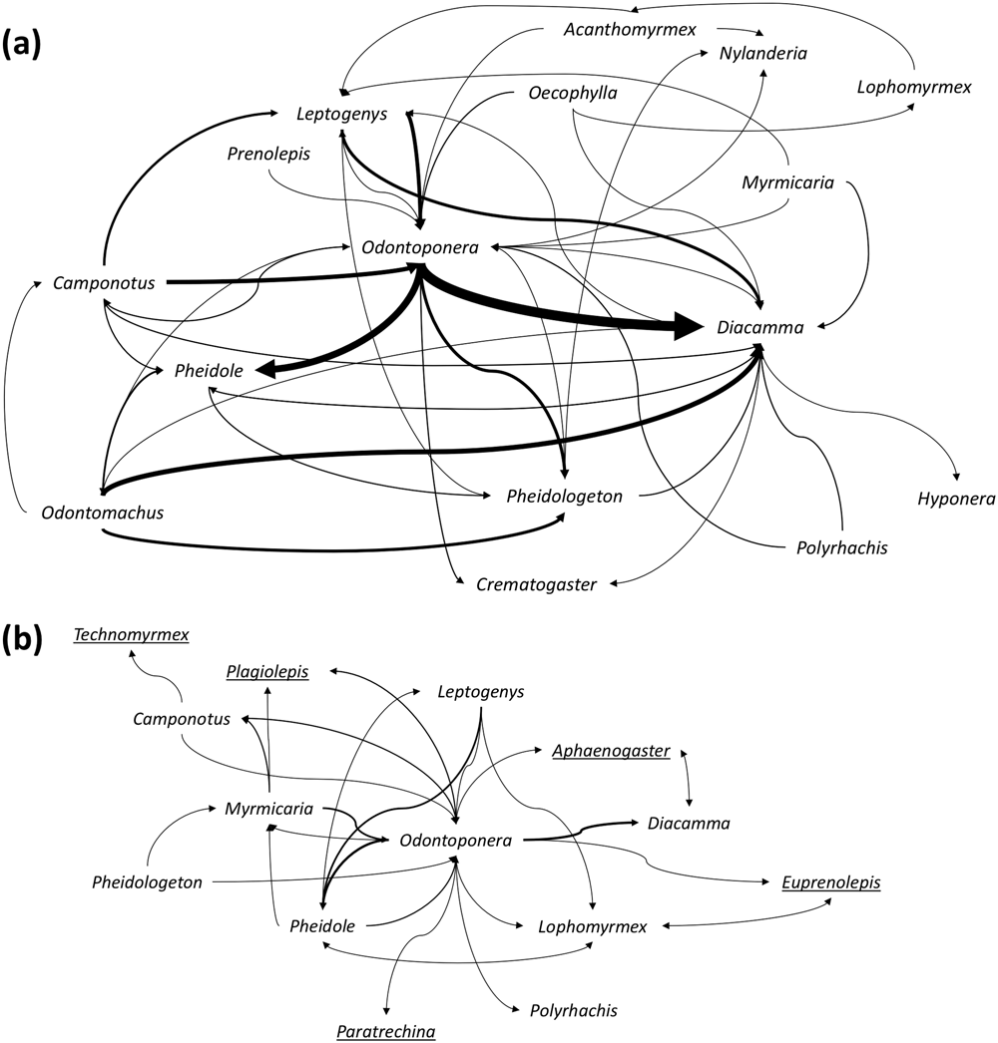
Interaction networks of genera in (**a**) primary and (**b**) logged forest. Only the aggressive ‘face-to-face’ interactions on a bait card are shown, represented by arrows pointing towards the overall submissive genus, with width indicating the number of aggressive interactions towards that submissive genus; thinnest 0.5 pt and thickest 8 pt lines indicative of 1 and 16 observed interactions respectively. Underlined genera indicate new arrivals to the logged network which were not previously seen in the primary network.

**Figure 5:**
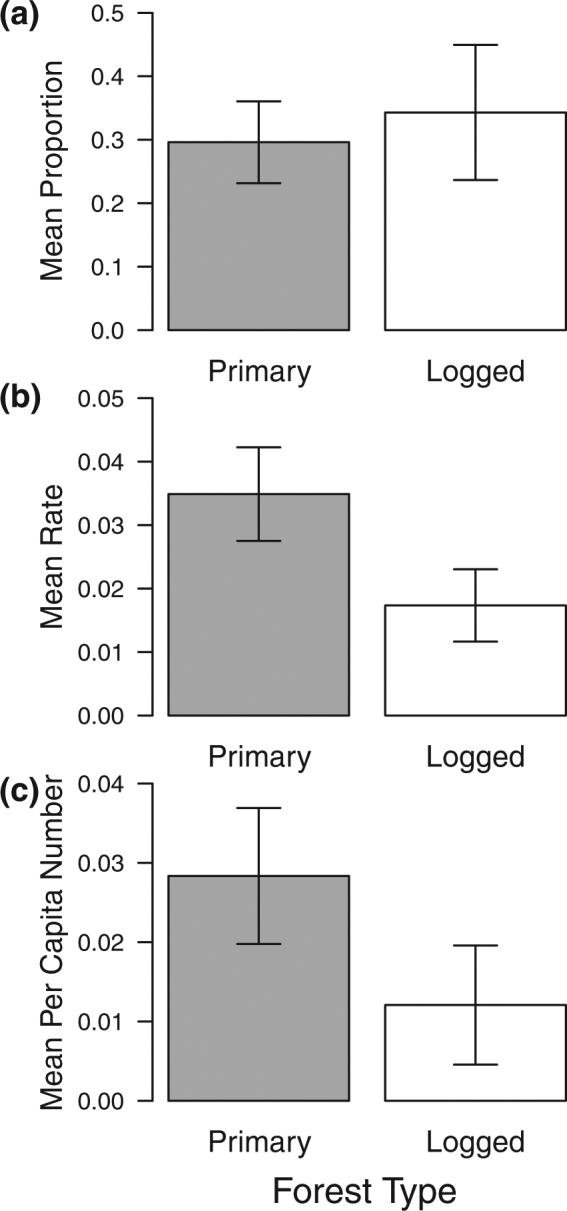
Competitive interactions observed at bait cards across the ant community differ between forest types. Mean (**a**) proportion out of total interactions (**b**) rate (number of interactions per 40 mins) (**c**) per capita number. Error bars represent 95% CIs.

The competitive network visually appeared simplified in logged relative to primary forest, with several pairwise interactions observed in primary forest not observed in logged forest (Fig. 4). The trend in the proportion of ant competitive interactions suggested a higher proportion in disturbed forest, but this was not significantly different between primary and logged forest (Mean: 0.296 vs. 0.343; GLMER: Z_147_ = 1.354, *P* = 0.176; Fig. 5a; Table S1). Both the rate and the per capita number of competitive interactions occurring at bait cards were significantly higher in primary than in logged forest (rate: mean = 0.035 vs 0.017 min^−1^, LMER: t_147_ = 3.775, *P* < 0.05; per capita: mean = 0.028 vs 0.012 per individual, LMER: t_147_ = 2.712, *P* < 0.05; Fig. 5b,c; Table S1).

**Figure 6:**
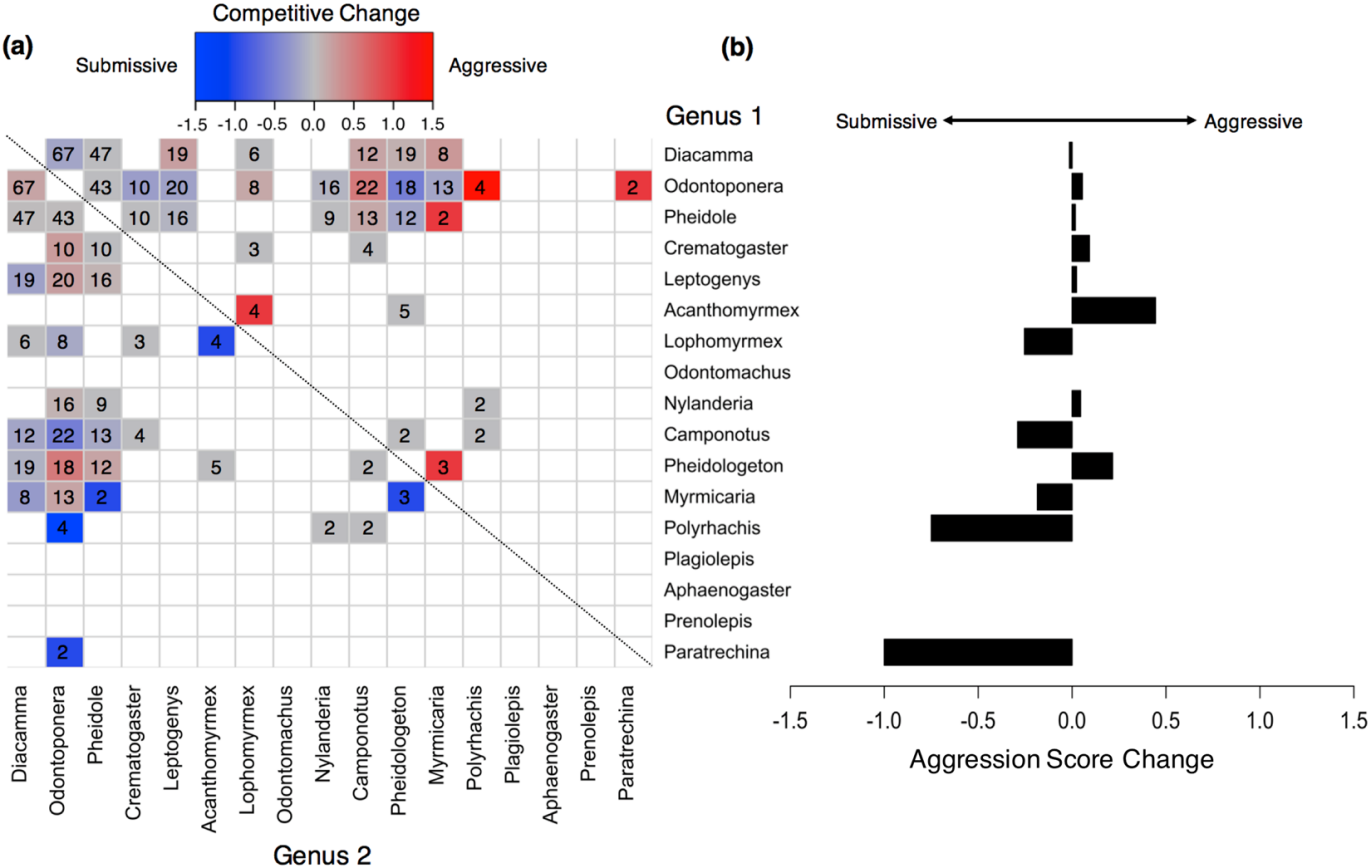
(**a**) Pairwise comparisons showing the change from primary to selectively-logged forest in mean aggression-scores for each genus-genus interaction. Direction of interaction is from genus 1 to genus 2. Colour gradient represents a gradient of change in competitive score: warm colours (towards red) = more aggressive (>0), grey = no change (0), colder colours (towards blue) = more submissive (<0). White squares indicate missing values due to less than three pairwise interactions being observed. Numbers in each box show sample size. (**b**) Bar graph showing the mean change in aggression-score for each genus across all interactions observed. Means are weighted based on sample size.

**Figure 7:**
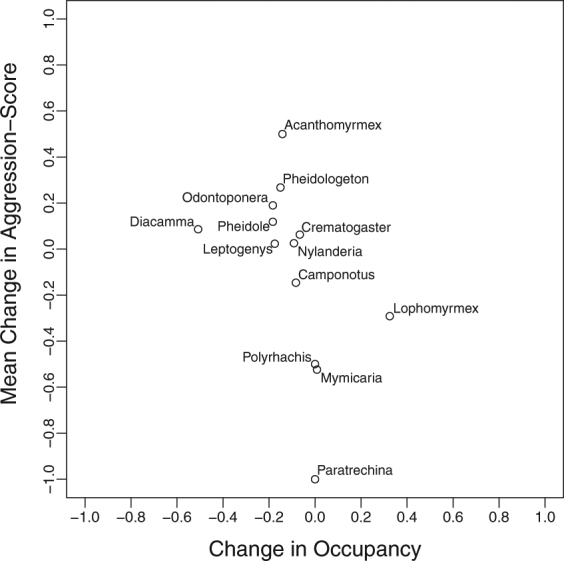
Plot displaying the relationship between change in occupancy of each genus and their mean change in aggression-score. For the mean change in competitive score >0 = more aggressive, 0 = neutral and <0 = more submissive.

Our null expectation was that genera shared between the primary and logged forest will show a consistent pattern of pair-wise dominance for any interactions observed. However, we found a number of genera that showed a decrease (*Lophomyrmex*, *Myrmicaria* and *Polyrhachis*) or increase in aggression-score (*Acanthomyrmex*, *Odontoponera, Pheidologeton*) in the logged relative to primary forest (Figs 4 and 6; Fig. S3). Furthermore, change in rank occupancy did not appear to explain these observed differences as we found no correlation between mean change in aggression-score and change in rank occupancy (*S* = 462, rho = −0.269, *P* = 0.374; Fig. 7).

Smaller genera such as *Pheidole* and *Nylanderia* saw only minor changes in their mean aggression-scores (0.063 and 0.025 respectively; Figs 6 and 7; S4 and S5) and these genera remained high in rank occupancy in primary and logged forest (respectively: rank 2 remaining 2, and rank 4 remaining 4; Fig. 3). By contrast, the particularly large dominant genera of *Odontomachus* and *Oecophylla* were absent in most leaf litter samples of logged forest, which matched average declines in the competitive score of the larger ant genera (>20 mm) between forest types (Mean score change: −0.323). However, many genera showed contrary patterns of competitive score and occupancy changes, displaying idiosyncratic patterns (Fig. 7). For example, *Odontoponera* became considerably more dominant over *Polyrhachis* and *Camponotus* in logged than in primary forest (Mean: 0.189; Figs 6a and S3), despite having a lower occupancy (−0.183; Fig. 7). By contrast, *Lophomyrmex* became less dominant relative to *Acanthomyrmex* (−0.291 vs. 0.500; Figs 6 and S5), despite showing an increase in occupancy that was >2× greater than that of *Acanthomyrmex* (0.325 vs. −0.141; Fig. 7). *Myrmicaria* became more submissive in logged forest (−0.524; Figs 6 and S5), especially to *Pheidole* and *Pheidologeton*, yet saw almost no change in occupancy (+0.008; Fig. 7).

## Discussion

Our findings can be split into two main components. First, we found a difference in ant community composition between forest types, with lower species richness and community evenness in the logged compared to primary forest. This contributes to the growing evidence base that anthropogenically disturbed habitats may lead to reductions in invertebrate taxa^57,66,82,88–90^, and more broadly to reductions in biodiversity with alterations to animal communities^4,5,7^. Loss of invertebrate biodiversity is of concern considering the indirect impact on ecosystem processes^8,91^, particularly in the Indo-Malayan region where our study site is located, which has been predicted to lose up to 42% of biodiversity by 2100^26^. Second, we provide a novel insight into the relationship between variation in shared ant community and difference in the competitive interactions; considered to influence community structuring which in turn underpins key ecosystem functions^92^. Differences in the aggression-scores of genera shared between forest types meant that we could reject our null hypotheses, yet this did not appear to relate with occupancy change as no positive correlation was found, meaning we also must reject our so-called intuitive hypothesis. Instead, we found that an aggression-score change showed no correlation with occupancy change across genera and some genera showed an inverse relationship. Together this suggests a shuffling of apparent aggressive behaviour and could be considered a re-wiring of the competitive network after disturbance.

### Community composition differences

The change in community composition between the forest types in our study are consistent with previous studies showing lower ant diversity in logged forest^56,57^ and genus-specific differences in tolerance to forest disturbance^38,54,93,94^. Differences in tolerance of ground-dwelling ants to disturbance may be mediated by microclimate variations and be contributing to variation in community composition alongside competition^67,79,95–98^, especially since ground temperatures have been reported to be higher in the logged versus primary forest areas we studied^99^. Body size determines the constraints to which insects such as ants can perform under varying temperatures, including measures of competitive ability^24^. For instance, large ants have been found to be less abundant in logged forest^8,100^, and ant groups have been shown to competitively exclude other groups of similar sizes^24^.

### Community competitive interactions

The rate and the per-capita number of competitive interactions was significantly lower in the logged compared to primary forest, indicating a net reduction in competitive intensity in logged forest. Yet in contrast, a previous study in a temperate region showed that more open habitats increased rather than decreased competitive intensity^70^. A decrease in vegetation structure from logging^101^, is likely to alter the niches that ants have evolved to exploit and therefore may have effects on the performance of ants arriving and exploiting the bait cards, that are difficult to predict. Indeed, predicting the direction of effect on competition in diverse community networks, even at the genera level, is tough^71^. Resultantly, the development of general rules to predict competition intensity over a spatial and temporal scale under varied climatic and environmental conditions and stochastic processes remains a significant challenge.

Notably though, despite several previous studies observing and reporting competitive interactions within ant communities^11,24,41,42,70^, we show that the majority of interactions between ant genera were non-antagonistic, suggesting that neutral relationships between litter ant genera are common in both forest types, especially in primary forest. The previous studies highlighting frequent competitive interactions have been typically based on canopy ant communities, which could highlight that different functional guilds of ants defended resources in different ways – either through close contact stings and bites or through larger spatial scale chemical defence^102^. Differences which may allow lower levels of competitive interactions to be observed in the litter ant community^102^.

### Changes in competitive interactions among genera

The change in aggression-scores across genera between the logged and primary forest showed little consistency in the relationship with rank occupancy, aligning more with an idiosyncratic pattern. Previous studies have suggested that compositional changes (abundance and occupancy) may alter the dominance of a genus or alter the position of species in an interactive network in ants^49^ or in bacterial communities^103^. Whilst we standby the view that competition for food is an important process structuring communities^41^, our results do suggest that a number of other interacting factors could be having a large effect, and these may even be other components of competitive behaviour such as the discovery-dominance trade off that considers arrival time to a food resource^73,104^.

The idiosyncratic relationship we observed could be due to a form of dual process where the potential cascading effects of community compositional change, through physical changes to the environment, allows for a competitive advantage through an increase in overall competitive ability or competitive release, through changes in the competitive network, or vice versa^41,43,95^. Previous data showing idiosyncratic type responses of plant and animal communities to disturbance supports this^12,105^. For instance, in the logged forest, the reduction in occupancy of predatory and dominant genera like *Odontomachus* could have weakened any competitive exclusion they placed on other genera in the community, allowing others such as *Odontoponera* to increase in aggressive activities and thus dominate a resource. Nevertheless, we may not have detected a distinct change in aggression-score as a result of this cascading process. The reduced occupancy of these genera in logged forest may be due to equal declines in their food resources (e.g. termites) because of changes in vegetation structure and climate^82^. Previous studies showing lower predatory genera abundances in logged forest^8^, along with competitive release following the removal of dominant ant species supports the trends seen here^51,106,107^. Competitive release in logged forest may also explain why we see decreases in genus richness, given dominant species have been shown to promote species richness^49^.

The change in environment between forest types may further induce cascading effects, with idiosyncratic changes in aggression between genera being seen as a result of competitive disadvantages^43,95^. In primary forest, *Lophomyrmex* shows aggressive dominance over *Acanthomyrmex* but in logged the roles are reversed. This respective switch in dominance is most likely a result of them competing for the same niche space in each forest type, as they are considered functionally equivalent^82^ and interestingly are the same size^24^. However, as the environment changes, different traits may become advantageous and the competitive status is reversed. The decrease in aggression-score of *Lophomyrmex* and *Myrmicaria* could also be explained by their increase in occupancy, allowing for greater opportunity to compete and be aggressively dominated in logged forest. Notably, genera that saw few changes in their score such as *Pheidole* or *Nylanderia* may be a result of their subdominant position in the competitive hierarchy in the community allowing them to still compete for patchy resources and remain relatively high in occupancy, despite changes in the genus showing high aggression or disturbance effects^41,49,108^.

In conclusion, we provide a new insight into how a competitive network can change as a result of disturbance which helps us to better understand the mechanistic processes that reinforce the difference in ‘end-point’ community structure. Future studies can also look at how altered competitive processes may impact on ecosystem function given the important functional role of ants in tropical forests, such as scavenging, seed dispersal, predation and soil turnover^32^.

### Data availability

The data used in this study is available on Zenodo (10.5281/zenodo.1198302) and can also be accessed through the S.A.F.E. project website (www.safeproject.net; Dataset ID: 1).

## Electronic supplementary material

Supplementary Information
