# Supplementary material for: Effect of tropical forest disturbance on the competitive interactions within a diverse ant community
Source: Sci Rep. 2018 Mar 23;8:5131. doi: 10.1038/s41598-018-23272-y (PMC5865194; doi:10.1038/s41598-018-23272-y)
Supplement: Supplementary file 1 — Supplementary Information [file 41598_2018_23272_MOESM1_ESM.pdf]

# Effect of tropical forest disturbance on the competitive interactions within a diverse ant community

**Ross E. J. Gray, Robert M. Ewers, Michael J. W. Boyle, Arthur Y. C. Chung and Richard J. Gill**

## Supplementary Methods

When placing sampling sites along the transect for both forest types, some sites were shifted away from the line due to obstacles such as rivers or fallen trees. We note that during the study, there was an El Niño Southern Oscillation (ENSO) event, however, a previous study has shown there to be little annual variation in ant communities even in El Niño years which means that this phenomenon should not have provided any extreme results[1]. GoPro™ specifications were as followed: GoPro HERO3, 1080p @ 30 fps, ultra-sharp f/2.8-6 element lens, medium Field of Vision (GoPro Inc, USA). Quadrats ($1m^2$) were used as the size is considered standard practice for the Winkler bag extraction method[2].

To examine whether the genera in the competition data were representative of the whole ant community (Winkler bag data), Bray-Curtis dissimilarity indices were calculated[3]. A non-metric multidimensional scaling (NMDS) ordination was then used to determine the extent of any compositional dissimilarities between data sets[4]. The statistical process was repeated to examine the community similarities between primary and logged forest (Fig. S1).

From the column 2 video recordings, we assigned each genus to a body size category using the grid squares on the bait card as a scale (small = < 2mm; medium = 2-10mm; large =

> 10mm), as previous studies have shown that habitat differences can promote differences in body size[5] and body size differences can drive competitive community assembly[6]. For the analyses of the body size of genera we original placed each genus into one of five size categories (v. small = < 2mm; small = 2-4mm; medium = 4-10mm; large = 10-20mm; v. large = > 20mm), however due to low sample sizes in the small and v.large categories we pooled genera into new categories in which v.small became 'small' (<2mm), small and medium became 'medium' (2-10mm) and large and v. large became 'large' (>10mm) (see Figures S2 & S3 for comparisons in pooling).

When addressing the effect of body size on occupancy and mean aggression-score change using Kruskal-Wallis rank sum tests, we considered both the minimum and maximum body size observed for each genus as intra-genus variation in body size can be found.[7]

# Supplementary Results and Discussion

Fifty-four genera were found in the Winkler bags and camera observations but when including the human observed bait cards there were 62. However, these bait cards were only used to help identify the camera data and so the additional genera were excluded from the results. Preliminary analysis found that the genus richness data was over-dispersed and non-normally distributed so generalised linear models were used in the analysis (Shapiro: $W = 0.880$, $P < 0.001$).

Comparing the occupancy of each size category, there was a general increase in occupancy as the minimum and maximum genera body size increased (Fig. S2a), and whilst there appeared to be a trend for a higher occupancy of large genera in primary compared to logged forest we found no significant difference in all three size categories at both extremes (Minimum: Small: $W = 927$, $P = 0.687$; Medium: $W = 96.5$, $P = 0.962$; Large: $W = 9.5$, $P =$

0.199. Maximum: Small: W = 171.5, $P = 0.770$; Medium: W = 638, $P = 0.766$; Large: W = 24.5, $P = 0.170$). Interestingly, this means that same size ants are continuing to occupy logged forest despite potential increases in ground temperature. The trends seen here may be due to methodological issues biasing the size of ants caught. Bait traps appeared to generally attract larger, more mobile individuals (Fig. S1) and so the cameras may not pick up the increases in very 'small' ant genera. Alternatively, there is intra- and inter-species variation in worker sizes of ants which may not translate to a higher average size of each genus due differences in the degree of variation within a genus[7]. However, we found the same trends in our results regardless of using minimum or maximum body categories, indicating the largest or smallest individual of a genus was not biasing the statements we originally suggested. Further research is thus needed to assess whether this is reflected on a continuous size scale or at the species level. Sampling method did have an effect on the size of genera seen, with a significantly higher occupancy of larger genera observed on the camera recordings than collected in the Winkler bags, even when looking at minimum and maximum size extremes (Minimum: W = 30.5, = $P < 0.001$; Maximum: W= 65.5, $P = 0.030$; Fig. S3).

In looking at whether size could contribute in explaining change in aggression-score, we found a trend towards the larger species in a genus maintaining their competitive score by showing less variation (coefficient of variation for minimum body size: small = 123, medium = 54.3, large = 106; maximum body size: medium = 96.1, large = 83.3; Fig. S4). Whilst not a strong relationship, this suggests larger ants are better at buffering the changes to the interactive competitive network. Although, given this pattern was found not to be statistically significant at both body size extremes when considering positive and negative values (Minimum: $\chi^2 = 0.038$, df = 2, $P = 0.981$; Maximum: $\chi^2 = 0.327$, df = 1, $P = 0.568$), and differences from zero (Minimum: $\chi^2 = 1.784$, df = 2, $P = 0.41$; Maximum: $\chi^2 = 0.619$, df = 1, $P = 0.431$), this requires further study.

# Supplementary Figures and Tables

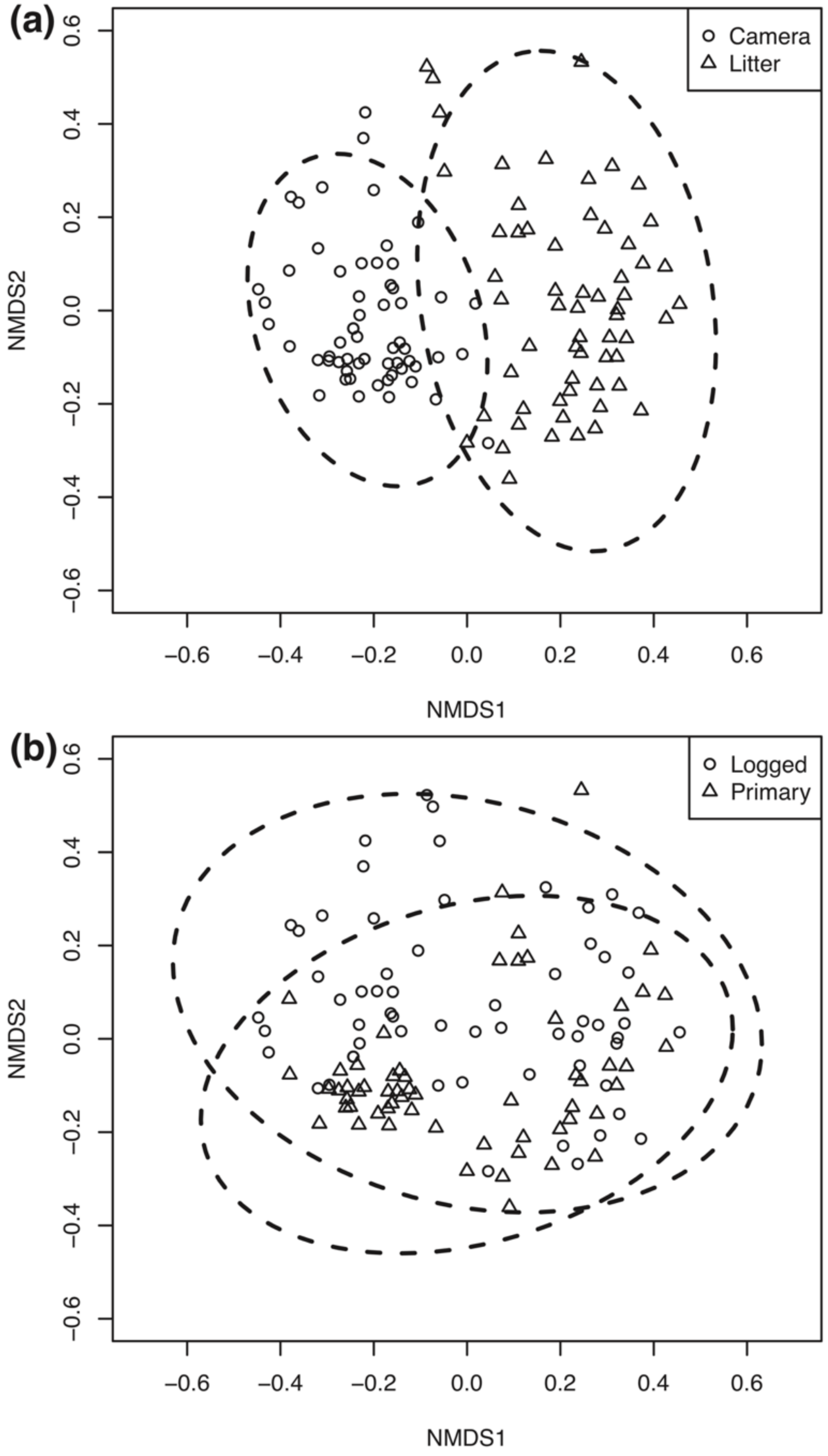

**Figure S1.** Non-Metric Multidimensional Scaling (NMDS) ordination showing the Bray-Curtis dissimilarity between genera communities. Ellipses represent the standard deviation for each community. (**a**) Circles represent the community video recorded on camera baits and triangles represent the community collected through leaf litter collection use Winkler bags. (**b**) Circles represent the community found in logged forest and triangles represent the community found in primary forest.

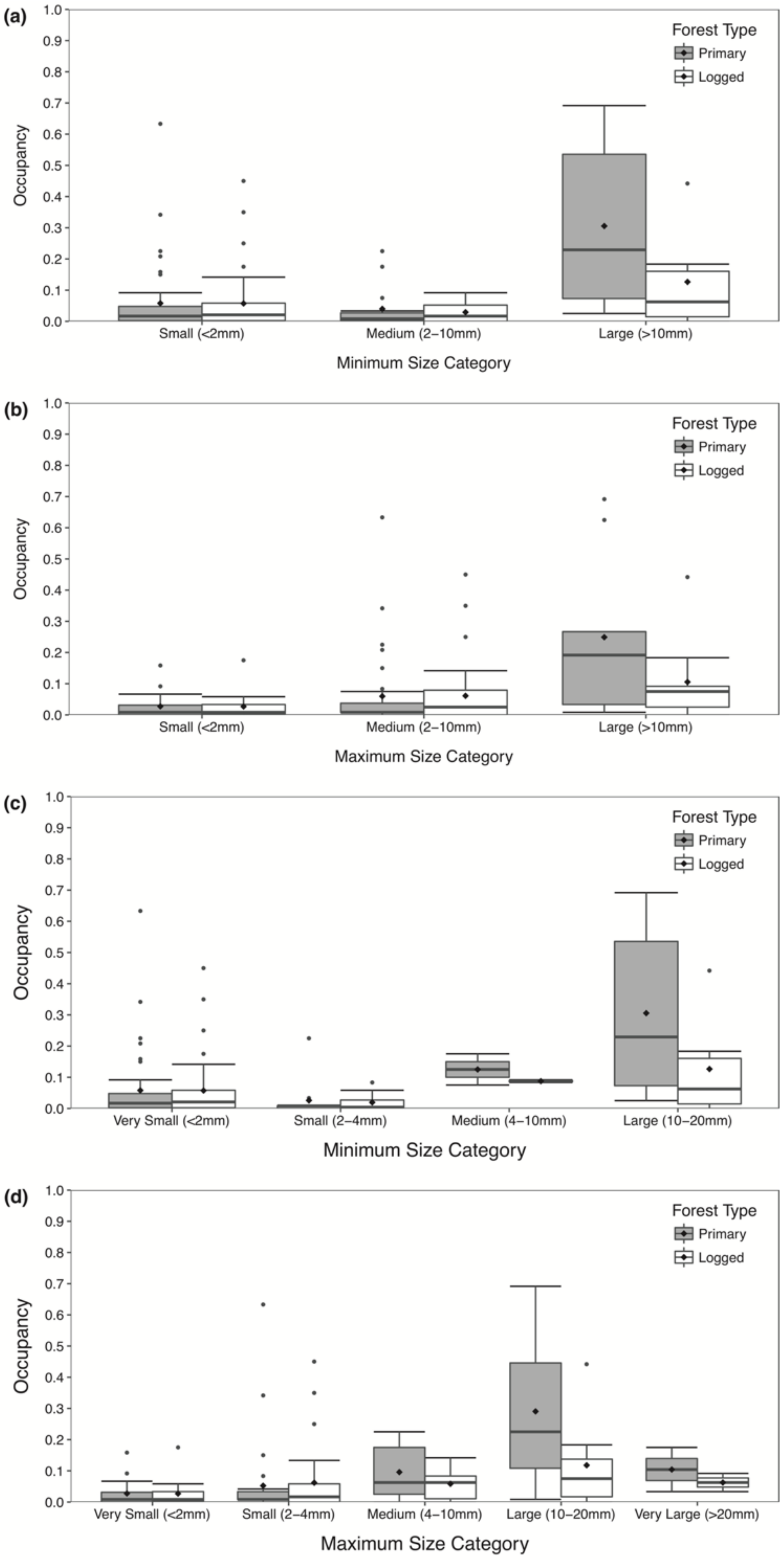
(a)
Occupancy
Forest Type
Primary
Logged
Small (<2mm)
Medium (2–10mm)
Large (>10mm)
Minimum Size Category
(b)
Occupancy
Forest Type
Primary
Logged
Small (<2mm)
Medium (2–10mm)
Large (>10mm)
Maximum Size Category
(c)
Occupancy
Forest Type
Primary
Logged
Very Small (<2mm)
Small (2–4mm)
Medium (4–10mm)
Large (10–20mm)
Minimum Size Category
(d)
Occupancy
Forest Type
Primary
Logged
Very Small (<2mm)
Small (2–4mm)
Medium (4–10mm)
Large (10–20mm)
Very Large (>20mm)
Maximum Size Category

82

**Figure S2.** Boxplots showing the occupancy of genera from different size categories in each forest type. Minimum size categories represent the minimum size category the individuals from each genus were recorded in and maximum represents the maximum size categories of recording. (**a**) and (**b**) show the pooled genera due to sample sizes and (**c**) and (**d**) the original five categories before pooling. Dissecting line and black diamond represents the median and mean, respectively, boxes represent the inter-quartile ranges, whiskers show 95% CI and dots show outliers.

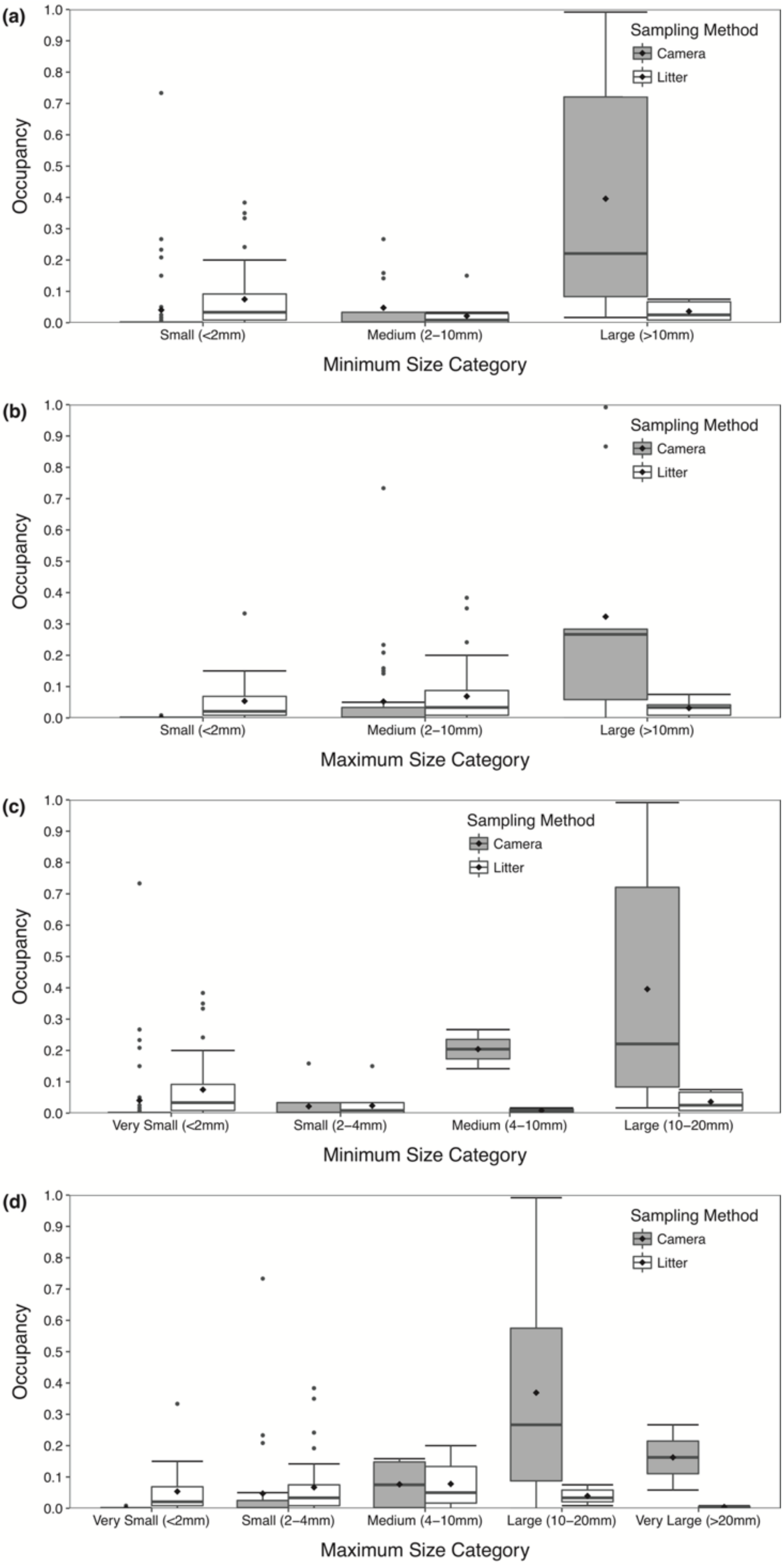
(a)
Occupancy
Sampling Method
Camera
Litter
Small (<2mm)
Medium (2–10mm)
Large (>10mm)
Minimum Size Category
(b)
Occupancy
Sampling Method
Camera
Litter
Small (<2mm)
Medium (2–10mm)
Large (>10mm)
Maximum Size Category
(c)
Occupancy
Sampling Method
Camera
Litter
Very Small (<2mm)
Small (2–4mm)
Medium (4–10mm)
Large (10–20mm)
Minimum Size Category
(d)
Occupancy
Sampling Method
Camera
Litter
Very Small (<2mm)
Small (2–4mm)
Medium (4–10mm)
Large (10–20mm)
Very Large (>20mm)
Maximum Size Category

**Figure S3**. Boxplots showing the occupancy of genera from different size categories collected in each sampling method. Minimum size categories represent the minimum size category the individuals from each genus were recorded in and maximum represents the same but with maximum size categories recorded. (**a**) and (**b**) show the pooled genera due to sample sizes and (**c**) and (**d**) the original five categories before pooling. Dissecting line and black diamond represents the median and mean, respectively, boxes represent the inter-quartile ranges, whiskers show 95% CI and dots show outliers.

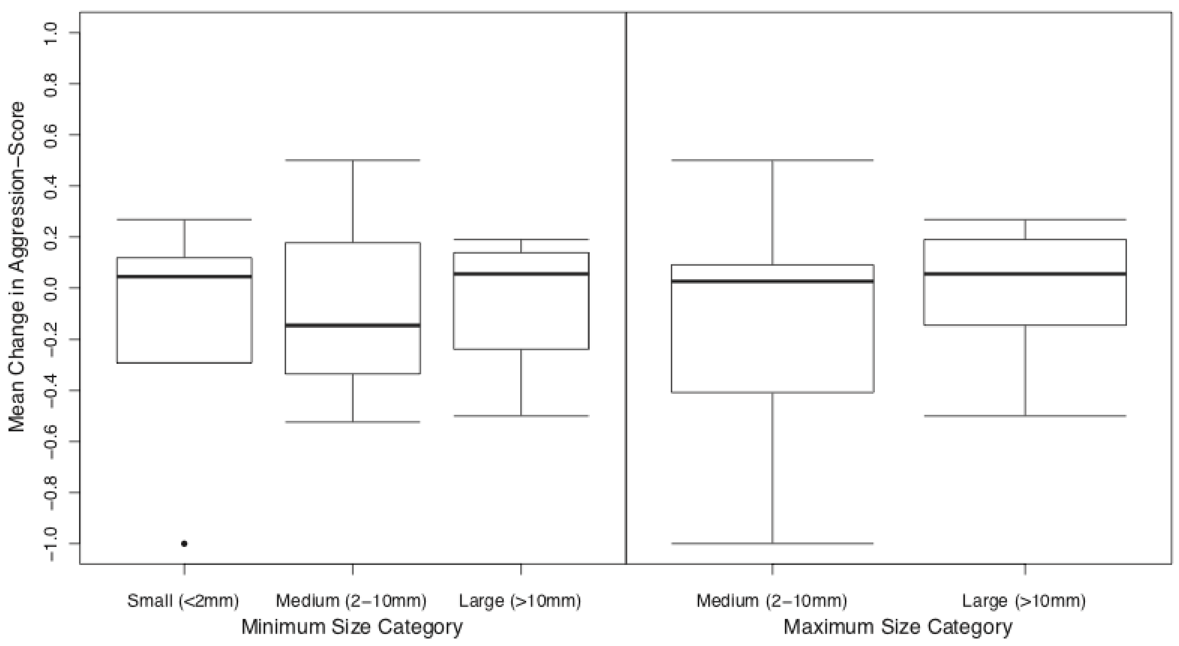


**Figure S4**. Plots displaying the different body size categories in relation to the mean change in aggression-score. For the mean change in aggression score > 0 = more aggressive, 0 = neutral and < 0 = more submissive. Minimum size category refers to the minimum size an individual from each genus was recorded in and maximum vice versa. The "small" category was excluded from the maximum size category panel due to lack of interacting genera at bait cards.

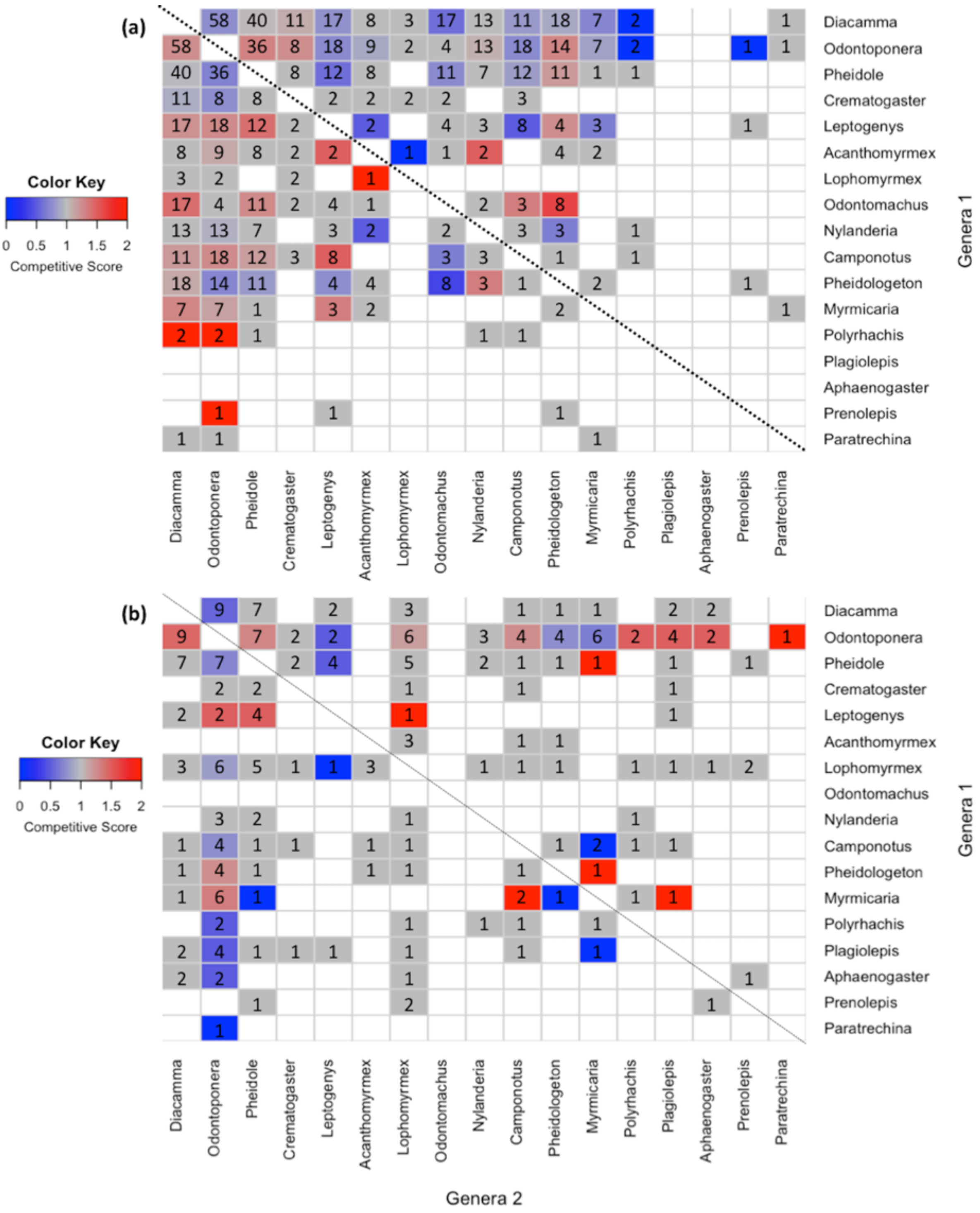


**Figure S5.** Pairwise comparisons for (**a**) primary and (**b**) selectively-logged forest showing the mean aggression-scores for each genus 1→ genus 2 interaction. Colour gradient represents a gradient of change in competitive score: warm colours (towards red) = more aggressive (> 0), grey = no change (0), colder colours (towards blue) = more submissive (<

0). White squares indicate missing values due to less than three pairwise interactions being observed. Numbers in each box show sample size.

**Table S1.** Summaries for the GLM and mixed effects models used in our analysis. The bottom three models refer to the proportion, rate and per capita number of competitive interactions occurring at a bait card. Site was included in the mixed-effect models as random effect.

| **Model** | **Variables** | **Slope** | **Standard Error** | **Z** | **df** | ***P*** | **Error Distribution** |
|---|---|---|---|---|---|---|---|
| GLM | Richness ~ Forest Type | 0.257 | 0.079 | 3.258 | 229 | 0.001 | Qaussipoisson |
| LM | Shannon Diversity ~ Forest Type | 0.184 | 0.067 | 2.759 | 118 | 0.007 | Guassian |
| GLM | Ratio ~ Forest Type | −0.411 | 0.216 | −1.89 | 17 | 0.059 | Binomial |
| GLMER | Proportion ~ Forest Type | −0.545 | 0.402 | −1.354 | 147 | 0.176 | Binomial |
| LMER | Rate ~ Forest Type | 0.018 | 0.005 | 3.775 | 147 | <0.05 | Gaussian |
| LMER | Per Capita ~ Forest Type | 0.016 | 0.006 | 2.712 | 147 | <0.05 | Gaussian |

# Supplementary References


1 Woodcock, P. *et al.* The conservation value of South East Asia's highly degraded forests: evidence from leaf-litter ants. *Philosophical Transactions of the Royal Society of London B: Biological Sciences* **366**, 3256-3264 (2011).

2 Krell, F.-T. *et al.* Quantitative extraction of macro-invertebrates from temperate and tropical leaf litter and soil: efficiency and time-dependent taxonomic biases of the Winkler extraction. *Pedobiologia* **49**, 175-186 (2005).

3 Bray, J. R. & Curtis, J. T. An ordination of the upland forest communities of southern Wisconsin. *Ecological monographs* **27**, 325-349 (1957).

4 Oksanen, J. *et al.* vegan: Community Ecology Package. R package version 2.4.3. (2013).

5 Kaspari, M. & Weiser, M. The size–grain hypothesis and interspecific scaling in ants. *Functional Ecology* **13**, 530-538 (1999).

6 Fayle, T. M., Eggleton, P., Manica, A., Yusah, K. M. & Foster, W. A. Experimentally testing and assessing the predictive power of species assembly rules for tropical canopy ants. *Ecology Letters* **18**, 254-262 (2015).

7 Hölldobler, B. & Wilson, E. O. *The ants*. (Harvard University Press, 1990).
